# A practical guide to estimating the light extinction coefficient with nonlinear models—a case study on maize

**DOI:** 10.1186/s13007-021-00753-2

**Published:** 2021-06-12

**Authors:** Josefina Lacasa, Trevor J. Hefley, María E. Otegui, Ignacio A. Ciampitti

**Affiliations:** 1 Department of Agronomy, Kansas State University, 1712 Claflin Rd, Manhattan, KS 66506 USA; 2 Dpto. de Producción Vegetal, Facultad de Agronomía, Universidad de Buenos Aires, Av. San Martín 4453 (C1417DSE), Ciudad de Buenos Aires, Argentina; 3 Department of Statistics, Kansas State University, 205 Dickens Hall, 1116 Mid-Campus Drive North, Manhattan, KS 66506 USA; 4 Consejo Nacional de Investigaciones Científicas y Técnicas (CONICET), Centro Regional Buenos Aires Norte, Estación Experimental Agropecuaria Pergamino INTA, Ruta 32 km 4.5, Pergamino (C2700), Buenos Aires, Argentina

**Keywords:** Non-linear models, Radiation interception, Light attenuation, Bayesian models, *Zea Mays* L.

## Abstract

**Background:**

The fraction of intercepted photosynthetically active radiation (fPARi) is typically described with a non-linear function of leaf area index (LAI) and *k*, the light extinction coefficient. The parameter *k* is used to make statistical inference, as an input into crop models, and for phenotyping. It may be estimated using a variety of statistical techniques that differ in assumptions, which ultimately influences the numerical value *k* and associated uncertainty estimates. A systematic search of peer-reviewed publications for maize (*Zea Mays* L.) revealed: (i) incompleteness in reported estimation techniques; and (ii) that most studies relied on dated techniques with unrealistic assumptions, such as log-transformed linear models (LogTLM) or normally distributed data. These findings suggest that knowledge of the variety and trade-offs among statistical estimation techniques is lacking, which hinders the use of modern approaches such as Bayesian estimation (BE) and techniques with appropriate assumptions, e.g. assuming beta-distributed data.

**Results:**

The parameter *k* was estimated for seven maize genotypes with five different methods: least squares estimation (LSE), LogTLM, maximum likelihood estimation (MLE) assuming normal distribution, MLE assuming beta distribution, and BE assuming beta distribution. Methods were compared according to the appropriateness for statistical inference, point estimates’ properties, and predictive performance. LogTLM produced the worst predictions for fPARi, whereas both LSE and MLE with normal distribution yielded unrealistic predictions (i.e. fPARi < 0 or > 1) and the greatest coefficients for *k*. Models with beta-distributed fPARi (either MLE or Bayesian) were recommended to obtain point estimates.

**Conclusion:**

Each estimation technique has underlying assumptions which may yield different estimates of *k* and change inference, like the magnitude and rankings among genotypes. Thus, for reproducibility, researchers must fully report the statistical model, assumptions, and estimation technique. LogTLMs are most frequently implemented, but should be avoided to estimate *k*. Modeling fPARi with a beta distribution was an absent practice in the literature but is recommended, applying either MLE or BE. This workflow and technique comparison can be applied to other plant canopy models, such as the vertical distribution of nitrogen, carbohydrates, photosynthesis, etc. Users should select the method balancing benefits and tradeoffs matching the purpose of the study.

**Supplementary Information:**

The online version contains supplementary material available at 10.1186/s13007-021-00753-2.

## Background

Crop growth models are useful tools to assist agronomists and farmers on their management decisions aimed to improve farming systems. These models rely on the estimation of light interception, as it is the source of energy for biomass production [1–4]. The underlying model for biomass production per unit land is a function of incident photosynthetically active radiation (PAR), the fraction of PAR intercepted by the canopy (fPARi), and the radiation use efficiency, i.e. biomass produced per unit of energy (RUE):

1 $$\mathrm{Biomass}=\mathrm{PAR}\cdot \mathrm{fPARi}\cdot \mathrm{RUE}.$$

The estimation of fPARi provides insights on the energy available for growth. The fPARi holds a nonlinear relationship with the leaf area index (LAI—crop leaf area per unit of land) described with the Beer–Lambert Equation [5–7]:

2 $$\mathrm{fPARi}= 1-{e}^{-k \mathrm{LAI}},$$

where fPARi (a proportion) is the response variable and takes values between 0 and 1, *k* is the light extinction coefficient of the crop, LAI is the predictor variable. The coefficient *k* partially defines the shape of the curve of fPARi versus LAI, i.e. the vertical light distribution. Lower values of *k* are related to higher levels of RUE since the uppermost leaf layer is not light-saturated, and the canopy is more efficient producing biomass with luminic energy [8]. In maize (*Zea mays* L.), *k* takes values between 0.4 and 0.7 at flowering stages [9, 10]. At a given time of the day and without nutrient or water deficits, *k* depends primarily on canopy structure defined by the combination of genotype, plant density and row spacing [11].

To obtain inference and accurate predictions from field data, scientists follow a series of steps. First, a mathematical model (Eq. 2) with unknown parameters (i.e. *k*) and a statistical model are formulated. Statistical models are needed to make inference from field data, because (i) the relationship between fPARi and LAI cannot be measured perfectly and (ii) the Beer–Lambert model is only a simplification of reality (i.e., the relationship between $\mathrm{fPARi}$ and LAI is not deterministic). As a result, statistical techniques must be used to estimate *k* from field data and there are many options with different assumptions and varying levels of inference (Table 1). Historically, the most common techniques to perform such a nonlinear regression were the least squares estimation (LSE), later replaced by maximum likelihood estimation (MLE) [12]. Bayesian estimation (BE) has not been applied yet to *k* estimation, but this method has demonstrated to be advantageous in other cases [13] and thus, it will be considered in the present study. For both MLE and BE, fPARi in Eq. (2) represents the expected value of a statistical distribution of the data (i.e. likelihood function). This distribution must be selected during model design and could be normal or beta, among others. Ideally, the model (i.e. the combination of the deterministic equation and the chosen likelihood functions) should be consistent with the underpinning plant process. For example, fPARi takes values between 0 and 1, thus a model that is used for prediction should predict values of fPARi between 0 and 1. Unfortunately, as we will discuss, models commonly used to estimate *k* do not always adhere to this and other important principals.

**Table 1:** Summary of the proposed techniques (LSE: least squares estimation, MLE: maximum likelihood estimation, LogTLM: log-transformed linear model using maximum likelihood estimation, and Bayesian) for the estimation of *k*, regarding their assumption level, availability of uncertainty estimates (e.g., standard errors, prediction intervals), tools for statistical inference (e.g., p-values, confidence intervals) and their estimate’s asymptotic properties

Technique	Response variable	Level of assumptions	Uncertainty estimates	Statistical inference	Unbiased estimates	Requires priors	Uses information from previous studies	Can be used to propagate uncertainty
LSE	fPARi	Minimum	No	No	Not applicable	No	No	No
MLE	fPARi	Intermediate	Yes	Yes	Yes	No	No	Yes
LogTLM	log(1-fPARi)	Intermediate	Yes	Yes	Yes	No	No	No
Bayesian	fPARi	Maximum	Yes	Yes	Depends	Yes	Depends	Yes

Alternatively, the response variable in Eq. (2) can be log-transformed to obtain a linear equation:

3 $$\mathrm{log}\left(1-\mathrm{fPARi}\right)= -k\cdot LAI.$$

It is important to note that such a transformation changes the assumptions and hence, the model and the results (e.g. values of *k* and predictions of fPARi). For example, if we assumed a normal distribution for both models, Eq. (2) would have an additive, normally distributed error $\varepsilon$ of fPARi. In contrast, re-transforming the response to the observation-level in Eq. (3) (i.e. fPARi and not the logarithm), the error would act multiplicatively (i.e. ${e}^{\varepsilon }$) and have a log-normal distribution. A common practice in the literature (review analysis, Additional file 1: Table S1, [14–42]) is to estimate *k* with a log-transformed linear model (LogTLM, Eq. 3) but then, use that estimate for predictions in models like Eq. (2). In other words, the coefficient *k* of a LogTLM is optimal for that model (Eq. 3) that uses log(1-fPARi) but not for the prediction model (Eq. 2), that uses fPARi (i.e. there is a better estimate for *k* at the observation level). These practices lack consistency because they combine different models in the estimation and prediction and should be avoided.

It is important to choose an appropriate statistical estimation technique that matches the goals of the study because each approach will produce different results due to different underlying assumptions. Thus, knowing the benefits and tradeoffs of the alternatives is crucial. Currently, studies in the literature mostly report (i) LogTLM or (ii) perform nonlinear regressions assuming normality of the data (Fig. 2). Moreover, we suspect that models are sometimes reported incorrectly because the differences between models are overlooked (Additional file 1: Table S1). The objective of this study was to (i) review and contrast these statistical techniques and (ii) apply the techniques to field data with different models as to demonstrate the strengths and weaknesses of each method. In this sense, this can help as a guide for researchers who aim to estimate a non-linear parameter like the coefficient *k* and are not certain about which technique to use.

### Statistical methods

The parameters involved in a deterministic relationship may be estimated using a set of alternative methods. Their levels of assumptions will be directly related to the possible level of inference. In the current study, we focused on the most relevant statistical methods to evaluate this practical issue. Firstly, we applied LSE due to its frequent implementation and relevance before the introduction of MLE in the early 20th century [12]. Secondly, we presented the Frequentist approach, and lastly, we introduced the Bayesian methods due to the great rate of growth and potential there is among the applications of this statistical framework.

#### Least-squares estimation

**Fig. 1:**
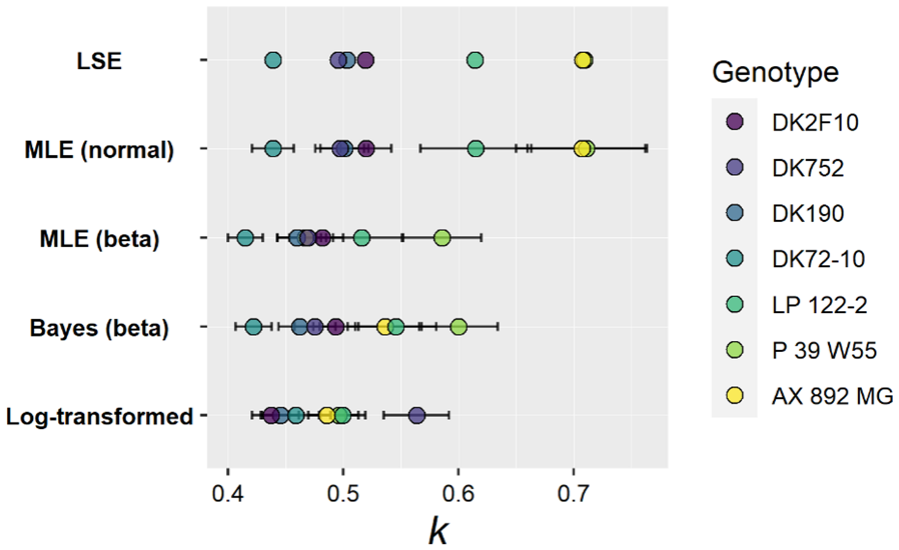
Technique comparisons. Points indicate point estimates or posterior means for the different implemented statistical techniques: Least squares estimation (LSE), maximum likelihood estimation (MLE) normal or beta distributions, log-transformed linear model and Bayesian (Bayes). Error bars indicate standard error

The LSE is considered a proper method to estimate parameters ‘objectively’ [43] but yields a single number, known as a point estimate, with no measures of uncertainty (Fig. 1). The LSE is considered “objective” because assumptions are minimal: the sum of squared errors (i.e. the loss function) is minimized and that is the only criterion to determine the best value for the estimate. A least squares estimate cannot have standard errors or confidence intervals because there is no statistical model associated with this technique, i.e. no assumptions about the data (e.g. normal distribution) are made. Consequently, point estimates may differ among genotypes, but with LSE one cannot obtain standard errors, confidence intervals or p-values to compare them, because that needs additional assumptions. This method alone would not be able to evaluate statistically significant differences between two canopy structures. The main inconvenience regarding choosing LSE is the lack of uncertainty estimates, which makes inference very limited.

Differences among canopy structures can only be inferred by assuming a likelihood function (e.g. assuming a normal distribution and applying MLE). Within the plant sciences literature, we have found examples of researchers reporting standard errors (or p-values) and claiming to use LSE [44, 45]. We suspect that those researchers were using MLE, a technique implemented in commonly used software (e.g., using the *nls* function in R [46] or similar nonlinear LSE along with the R function *confint*). However, the validity of additional assumptions (like the normal distribution of the data) should be tested. Other researchers have fit non-linear models to each replicate to estimate *k* and then performed ANOVA and post hoc tests, using those estimates as observations [47]. Nonetheless, this practice should be avoided since it does not account for estimation uncertainty and thus is more likely to find significant differences (p < 0.05) when none exist. In summary, using LSE cannot offer confidence intervals of the estimates; methods that do so might be using MLE and should assess whether the extra assumptions are valid.

#### Maximum likelihood estimation

The MLE is one of the most widely used statistical estimation techniques [48]. It treats the parameters as fixed variables, which may then be estimated using the data. The MLEs are expected to get very close to the ‘true value’ when sample size is large, (i.e. they are asymptotically unbiased), which is a desirable characteristic for scientists. Unbiasedness is an absent concept in LSE.

When assuming a normal distribution of the data and applying MLE, estimates are the same as LSE—but that does not hold for other distributions in MLE. First, LSE and MLE are sometimes used as synonyms because the point estimates are exactly the same if a normal distribution is assumed together with MLE. Hence, using a nonlinear mathematical model and obtaining confidence intervals is done by assuming a normal distribution of the data. However, this would require assessing the validity of the additional assumption. In fact, normality is not always the case: for variables with a limited range of values (e.g. proportions between 0 and 1), a normal distribution could produce unreasonable predictions or prediction intervals (i.e. < 0 or > 1). The likelihood function in MLE is selected during model design and can be different than normal [e.g. beta (continuous between 0 and 1), gamma (positive continuous), etc.]. In those cases, MLE and LSE will no longer be equal (Fig. 1).

#### Bayesian estimation

Bayesian statistics’ main difference to MLE is that it treats all unobserved quantities as random variables, according to Bayes’ Theorem:

4 $$P\left(\theta |y\right)=\frac{P\left(y|\theta \right) P(\theta )}{P(y)},$$

where $P(y|\theta )$ (likelihood) is the probability of observing the data given a deterministic model – it is the same likelihood used in MLE; $P\left(\theta \right)$ (prior) reflects the knowledge about the parameters before observing the data. $P(y)$ normalizes the joint distribution (likelihood × prior) so that the integral of the distribution integrates to 1. This is the reason why the posterior distribution is a probability distribution [49]. Thus, Bayesian statistics allows to make inferences based on probabilities [50].

Under some conditions, the maximum likelihood estimates are equal to the modes of Bayesian posteriors: this is the case when using flat, improper priors (i.e. the ‘previous knowledge’ includes all values from negative infinity to positive infinity), assuming the same likelihood function (i.e. distribution). After assuming a statistical distribution, using MLE means choosing flat, improper priors. Thus, the point estimates are the same as the modes of Bayesian posteriors with flat, improper priors: the joint distribution (Eq. 4) is identical. Then, for the same deterministic model (i.e. mathematical equation) and likelihood function, the point estimate of MLE and the mode of BE with flat, improper priors are the same. Differences might arise when adding more information to the priors.

**Fig. 2:**
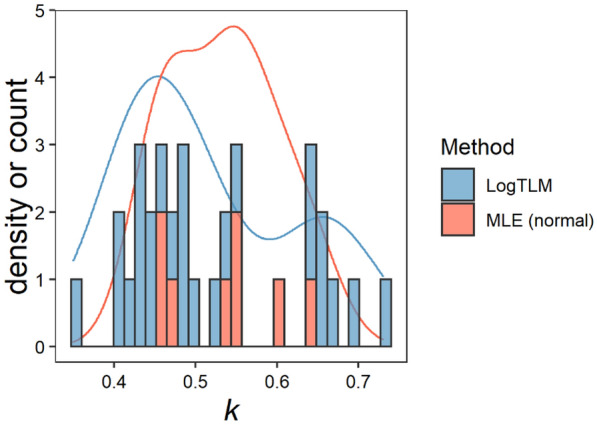
Summary of the values reported in the literature for the light extinction coefficient *k* in maize and their corresponding statistical method used in the estimation of this coefficient: maximum likelihood estimation for a log-transformed linear model [LogTLM], and maximum likelihood estimation with the assumption of normality [MLE (normal)]. Further details for the screening method can be found in Additional file 1: Table S1

An additional assumption and possible tradeoff of Bayesian statistics is the influence of the priors on the posterior (Eq. 4). Prior selection is an important step when designing a model, and may improve it by adding experts’ previous knowledge. As shown previously, designing the deterministic model and selecting the likelihood function also adds prior information (i.e. “subjectivity” or assumptions) to the model. Moreover, Bayesian statistics could include advances made in previous studies reflected in the priors [49]; especially for parameters like *k* that have been vastly studied (Fig. 2).

Moreover, large sample theory in Bayesian statistics demonstrates that posterior distributions of a parameter tend towards a single value (i.e. posterior consistency) [51]. This is similar to MLE asymptotic theory: the larger the number of observations, the narrower range of probable values for the parameter, until reaching a single point. Moreover, priors have less influence on the posterior for large sample sizes. This property of Bayesian statistics is desirable, considering the criterion of unbiased estimates.

#### Transformations

So far, the proposed estimation techniques were dealing with the nonlinear model described in Eq. (2), but the most common technique is taking the natural logarithm of the response (Eq. 3) to obtain a linear model (Fig. 2). Although transformations can be useful, they change the assumptions of MLE and the numerical results are different because the model is different (Fig. 1). Sometimes, LogTLM fit the data better, but each case should be assessed individually since the distribution of the data will determine which method is valid [52]. As explained previously: Eqs. (2) and (3) are different models. Transformations could be implemented using either method (LSE, MLE or BE), but we have only included MLE in this analysis as an example.

## Materials and methods

### Experimental design

Two field experiments were conducted during the 2007/2008 (Exp1) and 2014/2015 (Exp2) growing seasons. Both were located in the Agricultural Station of INTA located at Pergamino (33º56′S, 60º34′W), Buenos Aires, Argentina which has silty clay loam soils (Typic Argiudoll). Exp1 was configurated in a split-split-plot design with three replications, with row spacing (70 and 52 cm) as the main factor, planting density (9 and 12 plants·m^−2^) as the second, and genotype (Nidera AX 892 MG, Pioneer 39W55 and LP 122–2) as the last. Exp2 was configurated in a split-plot design with three repetitions, with planting density (9 and 12 plants·m^−2^) as the main factor and genotype as the second factor. Both experiments had the same row orientation (NE-SW). The genotypes differed in their year of release: DK2F10 (1980), DK752 (1993), DK190 (2002) and DK72-10 (2012).

### Canopy architecture measurements

**Fig. 3:**
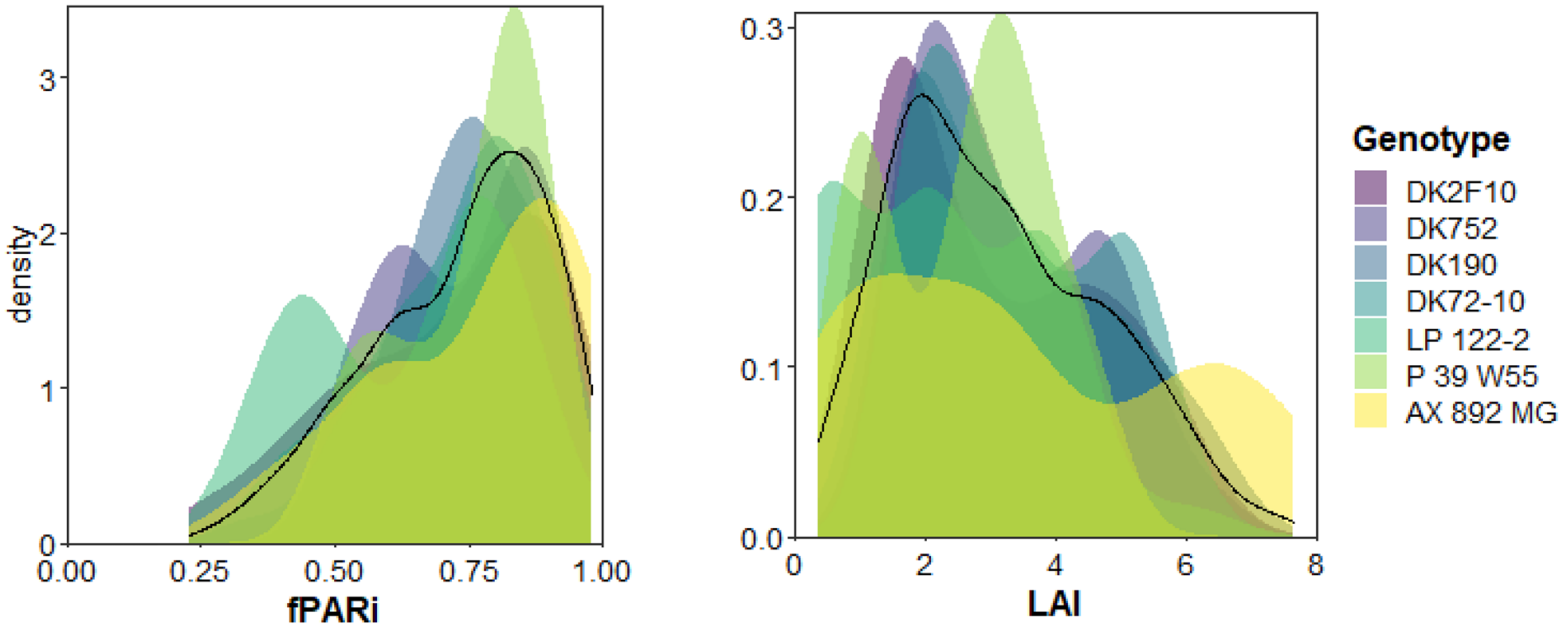
Data structure. Distribution of the observed values of the fraction of intercepted photosynthetically active radiation (fPARi) and leaf area index (LAI) for the whole data set (black line) and each genotype individually (colored distributions)

Individual leaf area was estimated using lamina length (L) and maximum width (W) [53] from six plants per plot:

5 $$Leaf\,Area = \alpha \cdot L \cdot W,$$

where α = 0.75 [54]. Leaf Area Index (LAI) per plot was estimated using the mean leaf area per plant (the mean of the sum of individual leaves) and stand density. LAI values ranged from 0.3 to 7.6 (Fig. 3).

### Light attenuation

Photosynthetically Active Radiation (PAR) was measured at the top (PAR_0_) and at the bottom (PAR_i_) of the canopy for both experiments. In addition, PAR was measured at different levels inside the canopy; two levels in Exp. 1 (two leaves below and two leaves above the ear leaf) and one level in Exp. 2 (at the level of the leaf holding the ear). All measurements were taken by the same person, around noon, on clear days with a 1 m long quantum-sensor placed diagonally with respect to the plant rows, in order to capture a representative portion of the light transmitted to the ground, below the plant canopy. The fraction of intercepted radiation (fPARi) at each level of the canopy was calculated as 1 − (PAR_i_/PAR_0_) [6] and ranged from 0.23 to 0.98 (Fig. 3).

### Statistical analysis

A total of five different methods (as combination of statistical techniques and models) were implemented to estimate the light extinction coefficient, using LSE, MLE and Bayesian approaches (Table 2).

**Table 2:** Summary of the statistical methods used in the analysis, and the frequency of their implementation in the scientific literature (Additional file 1: Table S1)

Technique	Deterministic model	Statistical distribution	Frequency of implementation in the literature
Least squares	Equation (2)	–	Never
Maximum likelihood	Equation (2)	Normal	Sometimes
Maximum likelihood	Equation (2)	Beta	Never
Maximum likelihood	Equation (3)^a^	Normal	Mostly
Bayesian	Equation (2)	Beta	Never

^a^Log-transformed linear model (LogTLM)

A nonlinear model was fitted using LSE using the “BFGS” algorithm of the *optim* function in R software [46] using Eq. (7).

The statistical model behind MLE and BE can be written out generally as:

6 $${y}_{ij}\sim P\left({y}_{ij}|{\mu }_{ij}, \psi \right),$$

7 $${\mu }_{ij}=1-{e}^{-{k}_{j}\cdot {\text{LAI}}_{ij}},$$

where ${y}_{ij}$ is the i-th observation of genotype j, ${\mu }_{ij}$ is the expected value of ${y}_{ij}$, $\psi$ is the dispersion parameter (i.e. variability of the data), ${k}_{j}$ is the light extinction coefficient for genotype *j* and ${\text{LAI}}_{ij}$ is the value of LAI of the i-th observation and genotype *j*. The expression in Eq. (6) implies that $y$ (fPARi) may have different probability distributions. This is an assumption a scientist makes during model design, in order to be able to make statistical inference. Making the additional assumptions explicit, Eq. (6) can be rewritten as

8 $${y}_{ij}\sim \mathrm{N}\left({\mu }_{ij},{\upsigma }^{2}\right),$$

or

9 $${y}_{ij}\sim \mathrm{beta}\left({\mu }_{ij},\kappa \right),$$

where ${\mu }_{ij}$ is the expected value (Eq. 7), ${\sigma }^{2}$ is the variance, and $\kappa$ is the dispersion of the normal and beta distributions, respectively.

Recall that MLE are equal to LSE when assuming normally distributed data (Eq. 8) and that most statistical software uses the normal distribution as the default option. However, if the data follow a normal distribution, a prediction could yield values that are not reasonable for a ratio like fPARi (e.g. < 0 or > 1). Instead, modelling the response variable with a beta distribution (which can take only values between 0 and 1) accounts for the possible values more realistically.

The MLE was applied to fit the data to two models: one assuming normal (Eq. 8) and a second one assuming beta distribution (Eq. 9). The optimization algorithm was “BFGS” using random starting values between 0.2 and 0.8, based on the literature that reports values for k between 0.35 and 0.80 (Additional file 1: Table S1). Approximate variances for the MLE estimate of *k* were obtained by inverting the Hessian matrix. Standard errors for the MLEs of *k* were obtained by taking the square root of the approximate variance. Likewise, the standard errors for the MLE of *k* were also used to construct Wald‐type confidence intervals (CIs). When using MLE, all uncertainty estimates for the parameter *k* (e.g., variances, SE, CIs etc.) requires “large sample” assumptions [51, 55].

A Bayesian model was fitted assuming a beta distribution (Eq. 9) and weakly informative priors:

10 $${k}_{j}\sim {\text{uniform}}\left(\mathrm{0,2}\right),$$

11 $$\upkappa \sim {\text{gamma}}\left(\mathrm{24,2}\right).$$

Note that a uniform(0,2) distribution gives the same likelihood to all values between 0 and 2, but assumes that values *k* > 2 or *k* < 0 cannot occur.

Last, a linear model with a log-transformed response variable (Eq. 3) was fitted using the *lm* function in R [46].

For each statistical technique, the mean squared error (MSE) was calculated as $\frac{\sum_{i=1}^{n}{(\text{fPAR}{i}_{predicte{d}_{i}}-\text{fPAR}{i}_{observe{d}_{i}})}^{2} }{n}$, where $\text{fPAR}{i}_{predicte{d}_{i}}$ and $\text{fPAR}{i}_{observe{d}_{i}}$ are the predicted and observed values of fPARi of the i-th observation, and *n* is the total number of observations. For LogTLM, the predicted values for log(1-fPARi) were back-transformed to the observation level (i.e. fPARi), to make the MSE values comparable among techniques.

The statistical techniques were compared according to (i) their possibility for inference (e.g. estimating standard errors, confidence intervals, p-values, etc.), (ii) theoretical properties of the point estimates and (iii) mean squared error, as a measure of predictive performance.

## Results

The point estimates for LSE and MLE (normal) were the same, whereas they were different to each other for the rest of the techniques—MLE (beta), Bayesian (beta) and LogTLM (Fig. 1; Table 3).

**Table 3:** Variability in the estimates of *k* for different genotypes (i.e., *k*_*j*_), depending on the statistical technique—point estimates and Bayesian posterior means, and 95% confidence or credible intervals in parenthesis

Genotype	LSE	MLE (normal)	MLE (beta)	LogTLM	Bayesian
DK190	0.50	0.50 (0.46–0.54)	0.46 (0.43–0.49)	0.50 (0.46–0.53)	0.46 (0.43–0.50)
DK2F10	0.52	0.52 (0.48–0.56)	0.48 (0.45–0.52)	0.49 (0.45–0.52)	0.49 (0.46–0.53)
DK72-10	0.44	0.44 (0.41–0.47)	0.42 (0.39–0.44)	0.45 (0.41–0.48)	0.42 (0.39–0.45)
DK752	0.50	0.50 (0.46–0.54)	0.47 (0.44–0.50)	0.44 (0.41–0.47)	0.48 (0.44–0.51)
LP 122–2	0.61	0.62 (0.52–0.71)	0.52 (0.45–0.58)	0.46 (0.40–0.52)	0.55 (0.48–0.62)
P 39 W55	0.71	0.71 (0.61–0.81)	0.59 (0.52–0.65)	0.56 (0.51–0.62)	0.60 (0.54–0.67)
AX 892 MG	0.71	0.71 (0.60–0.82)	0.47 (0.42–0.52)	0.50 (0.46–0.54)	0.54 (0.47–0.60)

*LSE* least squares estimation, *MLE* (normal) maximum likelihood estimation assuming a normal distribution of the data, *MLE* (beta) maximum likelihood estimation assuming a beta distribution of the data, *LogTLM* log-transformed linear model, and Bayesian techniques

**Fig. 4:**
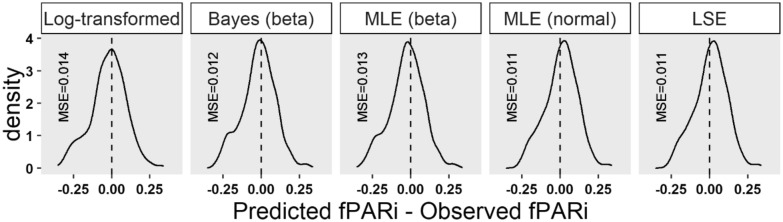
Empirical distributions of the difference between predicted and observed fPARi values for the five statistical methods, and the associated mean squared error (MSE). Note that in the log-transformed linear model, predictions had to be re-transformed to the observation level. MSE are defined as the mean squared difference between predicted and observed fPARi. *Log-transformed* log-transformed linear model, *Bayes (beta)* Bayesian model assuming a beta distribution of the data, *MLE* *(beta)* maximum likelihood estimation assuming a beta distribution of the data, *MLE* *(normal)* maximum likelihood estimation assuming a normal distribution of the data, *LSE* least squares estimation, techniques

The LogTLM was overall the most different method regarding the ranking and magnitude of *k,* and the predictive performance at the observation level (i.e. fPARi). Point estimates of *k* were lower for all genotypes and the ranking was notably different to the other methods (Fig. 1; Table 3). Moreover, when predictions were back-transformed to the observation level (i.e. fPARi instead of the logarithm), this method showed the highest mean squared error and a slightly worse residual distribution that either one of the nonlinear regression results (Fig. 4). The possible metrics for inference (i.e. standard errors, CI, etc.) were the same than nonlinear MLE; the main difference in choosing LogTLM lies in the value and ranking of *k*.

The LSE and MLE (normal) methods considered yielded similar residual distribution and mean squared error than MLE (beta) and BE; the main difference lies in the possibility for inference and model design. First, LSE point estimates are not compatible to make statistical inference. Second, MLE (normal) yields the same point estimates, and allows statistical inference and has asymptotically unbiased estimates. Note that the highest *k* estimates were given for LSE and MLE (normal).

## Discussion

This study compared LSE, MLE, LogTLM and BE methods to estimate a parameter (*k*) that is highly relevant for crop growth models and phenotyping. This research showcases the relevancy to report the statistical methods and assumptions used for its estimation because they have great influence on the numeric results, a critical step before comparing studies in the literature [56]. The LogTLM, reported in 76% of the screened studies (Additional file 1: Table S1), was substantially different from the non-linear models and performed the worst predicting fPARi at the observation level.

Changes in the results may affect interpretation and conclusions of a study, since *k* is related to several plant processes. Although results were consistent with the literature (Fig. 2), differences in magnitude and ranking among methods will lead to different conclusions. The plant processes explaining a same result in yield or biomass would thus differ [57, 58], since *k* is related to light interception and RUE [7, 8]. Possible consequences are (i) incorrect estimations of total PAR interception, leading to improper average RUE estimations, and (ii) incorrect inferences about vertical light and nitrogen distribution that affect photosynthesis and kernel set. Although small changes in magnitude may not affect total light interception when the plant canopy is closed (i.e. at high LAI levels), they may affect the physiological inferences. For example, differences in yield or kernel set between the hybrids DK190 and DK752 would be explained differently depending on the method used. First, using LSE or MLE (normal), their value of *k* is the same, and the differences might be explained e.g. with intrinsic efficiencies, rather than interactions with light distribution. However, using any other method, some differences might be explained by light or N distribution. It is important to keep this possible bias in mind when comparing *k* values from different studies [56] and before drawing conclusions from those studies. Choosing a statistical technique may be overlooked, but it could end up affecting the results, interpretation, and final conclusions of a study on this topic.

In addition, both LSE and MLE (normal) can return *k* values that may be considered comparatively high (in this case, > 0.70) (Additional file 1: Table S1). For instance, most studies in the literature report LogTLM, and therefore could be obtaining estimates with lower *k* values relative to LS estimates for the same data, as is the case in the current study. The light extinction coefficient of modern maize hybrids usually lies between 0.4 and 0.6 (Fig. 2). For example, a publication that implements LogTLM would report a value of 0.56 for the genotype P 39 W55. Further studies should obtain similar values for *k* for that particular canopy structure (i.e. combination of genotype, stand density and row spacing) because the coefficient *k* remains constant under potential conditions (i.e. well-watered and fertilized). However, the magnitude may change to 127% of LogTLM, only by changing the statistical technique. Thus, it could be plausible that studies facing similar problems never published their too-large estimates of k, if they only tried LSE methods [44, 59], and compared it to results from LogTLM.

All things considered, the LogTLM was less reliable in obtaining an adequate estimate of *k* and should be avoided for *k* estimation. This transformation into a linear model was very useful in times when the main restriction was computational power [60]. However, in the current study it presented the greatest MSE and distribution of the difference observed-predicted fPARi (Fig. 4). Similar cases can be found in the literature, where transformations of the data were the norm, but are outperformed by nonlinear regression techniques [13, 52, 61, 62]. At the early beginnings of *k* estimation in the 1950s [7], such a transformation was helpful. However, modern methods should be used to improve the estimation of this parameter and allow to make reliable comparisons between studies.

Furthermore, models with a beta distribution (applying either MLE or BE) can be pointed out as the most preferred over LSE and MLE (normal). First, although LSE can be a good choice for a single-point estimation, inference is not available. Simple research questions such as the existence of differences between genotypes cannot be answered with LSE methods. Instead, likelihood-based or Bayesian methods should be preferred because they allow the user to make statistical inference. Second, going directly from LSE to MLE implies assuming a normal distribution, when beta is the closest one to model reality: it produces values between 0 and 1, as expected for a proportion like fPARi. This common transition from LSE to MLE (normal) that can be found in the literature portrays Gelman and Hennig’s claim, when “Decisions that need to be made are taken out of the hand of the user and are made by the algorithm, removing an opportunity for manipulation but ignoring valuable information about the data and their background” [43]. In the current analysis, a beta is more appropriate than a normal distribution, whereas MLE and Bayesian are both adequate to obtain single point estimates. Bayesian estimates are similar to MLE and have shown to improve estimation for noisy data [63].

Bayesian techniques allow making probabilistic inferences and including expert’s prior information. First, BE provides entire posterior distributions instead of single point estimates and thus can be used to propagate uncertainty. Second, the MLE approach discards information learned in previous studies because it assumes “k lies somewhere in between negative infinity and positive infinity”. The Bayesian priors account for the state of knowledge about *k*, “it lies somewhere between 0 and 2”: something every researcher would agree on. Such information in the priors may reduce the amount of data required to achieve the same level of inference [64]. Additionally, previous studies have shown that Bayesian techniques are helpful to solve identifiability issues in MLE (e.g. with noisy data). Identifiability issues may arise when the data are sparse or present collinearity and magnify the uncertainty to estimate a parameter or a set of parameters. This can be avoided by using slightly more informative priors [65, 66]. Assumptions about *k* (priors) that are supported by previous works could be an advantage for improving inference efficiency or avoiding identifiability issues in MLE.

The current analysis can be understood as a case study for the estimation of a non-linear parameter: the light extinction coefficient *k*. The statistical method and comparisons can be directly applied to the same coefficient (to study similar processes) in other crops (e.g., sorghum—*Sorghum bicolor* (L.) Moech—or sunflower—*Helianthus annuus* L.). Other field methods should be explored to describe and quantify light attenuation due to changes in canopy architecture (e.g. soybeans, *Glycine max* L.). Additionally, this approach has great potential for modelling other variables that present vertical patterns in the canopy, i.e. nitrogen distribution, photosynthesis, carbohydrates, or other nutrients that follow the light attenuation canopy profile [67–72]. Nonlinear regressions like sigmoid growth curves or allometric relationships should be evaluated according to the data and its distribution [52, 73]: log-transformations are sometimes preferable [52, 73] or unrecommended [62]. In this study, both MLE (beta) and Bayesian methods provided robust models and yielded similar results. Further advantages from Bayesian statistics could be expected in other non-linear relationships that present identifiability issues in MLE [74], for parameters that in theory would need to be constrained as *k*, or adding complexity to the models.

Looking forward, new approaches integrating machine learning, remote sensing, and crop modeling may conform a proper complement to the current methodology to describe and quantify vertical canopy light distribution [75–78]. However, due to the advancements on these new methods in the last years, it is still surprising to find that only a few studies are applying these or any other new approaches tested for examining this critical factor affecting overall canopy photosynthesis and underpinning yield formation.

## Conclusion

This study provides a comparison to estimate the light extinction coefficient *k* using different methods (LSE, MLE, BE and LogTLM). The LogTLM has been the most frequently reported method but based on the results of this study it should be avoided for *k* estimation, mainly because (i) it yields the most different estimates compared to the other models, and (ii) the regression coefficients are optimal for models using the log-transformed variable, but suboptimal for models using the variable at the observation level (i.e. where predictions are required). Otherwise, the selected model and technique should match the purpose of the study, knowing benefits and tradeoffs. Since fPARi is a proportion that ranges from 0 to 1, models with a beta distribution instead of normal -currently absent in the literature- are more realistic and preferrable. In this case, selecting MLE or BE techniques, modelled with beta-distributed data was equally recommendable to obtain a single point estimate. The most popular approaches presented in the literature (LogTLM and MLE with normal distribution) are not adequate, and the new methods tested in this study (beta distribution applying MLE or BE) are highly recommended. This approach has the potential to be applied to other nonlinear regressions, such as the canopy distribution of nitrogen or other nutrients following light distribution.

## Supplementary Information

**Additional file 1: Table S1** Summary of values found in the literature for the light extinction coefficient *k* in maize and the statistical method used in the estimation. We conducted a search in the Web of Science database, using the search terms “corn/maize/Zea mays”, and “light extinction /light attenuation/light interception/extinction coefficient/attenuation coefficient”. From the resulting 422 publications, 35 were selected because they matched the following criteria: studies had to report estimates of k in maize, have plant densities between 6 and 12 plants m^−2^ (i.e. the same range as our experiments), and be written in English. There were no restrictions on date of publication. The mean vas selected for studies with treatments with several measurement moments [50], as well as the intermediate row spacing arrangements (i.e. 0.5-0.8 m) [51].

## Data Availability

The datasets analyzed during the current study are available from the corresponding author on reasonable request. R code is freely available at https://github.com/jlacasa/k-estimation/blob/main/k_estimation_02182021.Rmd.
