# Supplementary material for: A practical guide to estimating the light extinction coefficient with nonlinear models—a case study on maize
Source: Plant Methods. 2021 Jun 12;17:60. doi: 10.1186/s13007-021-00753-2 (PMC8196512; doi:10.1186/s13007-021-00753-2)
Supplement: Supplementary file 1 — Additional file 1: Table S1 Summary of values found in the literature for the light extinction coefficient k in maize and the statistical method used in the estimation. We conducted a search in the Web of Science database, using the search terms “corn/maize/Zea mays”, and “light extinction /light attenuation/light interception/extinction coefficient/attenuation coefficient”. From the resulting 422 publications, 35 were selected because they matched the following criteria: studies had to report estimates of k in maize, have plant densities between 6 and 12 plants m−2 (i.e. the same range as our experiments), and be written in English. There were no restrictions on date of publication. The mean vas selected for studies with treatments with several measurement moments [50], as well as the intermediate row spacing arrangements (i.e. 0.5-0.8 m) [51]. [file 13007_2021_753_MOESM1_ESM.docx]

**A practical guide to estimating the light extinction coefficient with nonlinear models – a case study on maize**

**Additional file 1**

Josefina Lacasa^1,2^, Trevor Hefley^3^, María E. Otegui^2,4^ and Ignacio Ciampitti^1^

^1^Department of Agronomy, Kansas State University, 1712 Claflin Rd, Manhattan, Kansas, 66506, US

^2^Dpto. de Producción Vegetal, Facultad de Agronomía, Universidad de Buenos Aires, Av. San Martín 4453 (C1417DSE), Ciudad de Buenos Aires, Argentina

^3^Department of Statistics, Kansas State University, 205 Dickens Hall, 1116 Mid-Campus Drive North, Manhattan, KS 66506, US

^4^Consejo Nacional de Investigaciones Científicas y Técnicas (CONICET), Centro Regional Buenos Aires Norte, Estación Experimental Agropecuaria Pergamino INTA, Ruta 32 km 4.5, Pergamino (C2700), Buenos Aires, Argentina

**Table S1**

Summary of values found in the literature for the light extinction coefficient *k* in maize and the statistical method used in the estimation. We conducted a search in the Web of Science database, using the search terms “corn/maize/Zea mays”, and “light extinction /light attenuation/light interception/extinction coefficient/attenuation coefficient”. From the resulting 422 publications, 35 were selected because they matched the following criteria: studies had to report estimates of *k* in maize, have plant densities between 6 and 12 plants m^-2^ (i.e. the same range as our experiments), and be written in English. There were no restrictions on date of publication. The mean vas selected for studies with treatments with several measurement moments [1], as well as the intermediate row spacing arrangements (i.e. 0.5-0.8 m) [2].

| Reference | *k* value | Reported statistical method |
| --- | --- | --- |
| Allen et al., 1962 | 0.70 | MLE log-transformed |
| McCaughey and Davies, 1974 | 0.43 | MLE log-transformed |
| Pepper et al., 1977 | 0.52-0.80 | MLE log-transformed |
| Spike and Tollefson, 1991 | 0.73 | MLE log-transformed |
| Tollenaar and Aguilera, 1992 | 0.65-0.74 | MLE log-transformed |
| Flénet et al., 1996 | 0.47-0.34 | MLE log-transformed |
| Maddonni and Otegui, 1996 | 0.46-0.64 | MLE with normal distribution* |
| Birch et al., 1999 | 0.46 | MLE with normal distribution |
| Maddonni et al., 2001 | 0.55-0.65 | MLE log-transformed |
| Tsubo et al., 2001 | 0.40-0.43 | MLE log-transformed |
| Cavero et al., 1999 | 0.49 | MLE log-transformed |
| Borrás et al., 2003 | 0.55-0.53 | MLE with normal distribution |
| Lizaso et al., 2003 | 0.40-0.70 | MLE log-transformed |
| Kiniry et al., 2004 | 0.47 | MLE log-transformed |
| Lindquist et al., 2005 | 0.67 | MLE log-transformed |
| D’Andrea et al., 2006 | 0.35-0.52 | MLE with normal distribution* |
| Maddonni et al., 2006 | 0.55 | MLE with normal distribution* |
| Awal et al., 2006 | 0.44 | MLE log-transformed |
| Drouet and Kiniry, 2008 | 0.30-0.40 | MLE log-transformed |
| Irmak and Mutiibwa, 2008 | 0.44 | MLE log-transformed |
| Bergamaschi et al., 2010 | 0.28-0.58 | MLE log-transformed |
| Vazin et al., 2010 | 0.65 | MLE log-transformed |
| Gao et al., 2010 | 0.46 | MLE log-transformed |
| Ma et al., 2014 | 0.46-0.62 | MLE log-transformed |
| Timlin et al., 2014 | 0.65 | MLE log-transformed*** |
| Wang et al., 2015 | 0.48 | MLE log-transformed |
| Morales-Ruiz et al., 2016 | 0.58-0.70 | MLE log-transformed |
| G. Liu et al., 2017 | 0.56-0.74 | MLE log-transformed |
| X. Liu et al., 2017 | 0.42 | MLE log-transformed |
| Soleymani, 2017 | 0.46-0.51 | MLE log-transformed |
| Dong et al., 2018 | 0.42-0.52 | MLE with normal distribution |
| Li et al., 2018 | 0.45-0.55 | MLE log-transformed |
| Perez et al., 2019 | 0.57-0.72 | MLE with normal distribution** |
| Kukal and Irmak, 2020 | 0.30-0.50 | MLE log-transformed |

** The paper reports a log-transformed function but fitted an exponential function.*

*** The paper reports LSE but provides standard deviations.*

**** The paper reports an exponential function but fitted a linear model.*

**References**

1. McCaughey JH, Davies JA. Diurnal variation in net radiation depletion within a corn crop. Boundary-Layer Meteorol. Springer; 1974;5:505–11.

2. Drouet J-L, Kiniry JR. Does spatial arrangement of 3D plants affect light transmission and extinction coefficient within maize crops? F Crop Res [Internet]. Elsevier; 2008 [cited 2020 Apr 6];107:62–9. Available from: https://www.sciencedirect.com/science/article/abs/pii/S0378429007002675

3. Allen LH, Yocum CS, Lemon ER. Radiant energy exchanges within a corn crop canopy and implications in water use efficiency. 1965.

4. Pepper GE, Pearce RB, Mock JJ. Leaf Orientation and Yield of Maize 1. Crop Sci [Internet]. Wiley; 1977 [cited 2020 Jun 24];17:883–6. Available from: https://acsess.onlinelibrary.wiley.com/doi/full/10.2135/cropsci1977.0011183X001700060017x

5. Spike BP, Tollefson JJ. Yield Response of Corn Subjected to Western Corn Root worm (Coleoptera: Chrysomelidae) Infestation and Lodging. J Econ Entomol [Internet]. 1991;84:1585–90. Available from: https://doi.org/10.1093/jee/84.5.1585

6. Tollenaar M, Aguilera A. Radiation Use Efficiency of an Old and a New Maize Hybrid. Agron J [Internet]. Wiley; 1992 [cited 2020 Jun 9];84:536–41. Available from: http://doi.wiley.com/10.2134/agronj1992.00021962008400030033x

7. Flénet F, Kiniry JR, Board JE, Westgate ME, Reicosky DC. Row spacing effects on light extinction coefficients of corn, sorghum, soybean, and sunflower. Agron J. 1996;88:185–90.

8. Maddonni GA, Otegui ME. Leaf area, light interception, and crop development in maize. F Crop Res. Elsevier B.V.; 1996;48:81–7.

9. Birch CJ, Hammer GL, Rickert KG. Dry matter accumulation and distribution in five cultivars of maize (Zea mays): relationships and procedures for use in crop modelling. Aust J Agric Res [Internet]. 1999;50:513–28. Available from: https://doi.org/10.1071/A98113

10. Maddonni GA, Otegui M., Cirilo A. Plant population density, row spacing and hybrid effects on maize canopy architecture and light attenuation. F Crop Res [Internet]. Elsevier; 2001 [cited 2019 Nov 22];71:183–93. Available from: https://www.sciencedirect.com/science/article/pii/S0378429001001587

11. Tsubo M, Walker S, Mukhala E. Comparisons of radiation use efficiency of mono-/inter-cropping systems with different row orientations. F Crop Res [Internet]. 2001;71:17–29. Available from: https://www.sciencedirect.com/science/article/pii/S0378429001001423

12. Cavero, Zaragoza, Suso, Pardo. Competition between maize and Datura stramonium in an irrigated field under semi-arid conditions. Weed Res [Internet]. John Wiley & Sons, Ltd; 1999;39:225–40. Available from: https://doi.org/10.1046/j.1365-3180.1999.00140.x

13. Borrás L, Maddonni GA, Otegui ME. Leaf senescence in maize hybrids: plant population, row spacing and kernel set effects. F Crop Res [Internet]. 2003;82:13–26. Available from: https://www.sciencedirect.com/science/article/pii/S0378429003000029

14. Lizaso JI, Batchelor WD, Westgate ME, Echarte L. Enhancing the ability of CERES-Maize to compute light capture. Agric Syst. 2003;76:293–311.

15. Kiniry JR, Bean B, Xie Y, Chen P. Maize yield potential: critical processes and simulation modeling in a high-yielding environment. Agric Syst [Internet]. 2004;82:45–56. Available from: http://www.sciencedirect.com/science/article/pii/S0308521X04000095

16. Lindquist JL, Arkebauer TJ, Walters DT, Cassman KG, Dobermann A. Maize Radiation Use Efficiency under Optimal Growth Conditions. Agron J [Internet]. John Wiley & Sons, Ltd; 2005 [cited 2020 Jun 27];97:72–8. Available from: http://doi.wiley.com/10.2134/agronj2005.0072

17. D’Andrea KE, Otegui ME, Cirilo AG, Eyhérabide G. Genotypic Variability in Morphological and Physiological Traits among Maize Inbred Lines—Nitrogen Responses. Crop Sci [Internet]. John Wiley & Sons, Ltd; 2006;46:1266–76. Available from: https://doi.org/10.2135/cropsci2005.07-0195

18. Maddonni GA, Cirilo AG, Otegui ME. Row Width and Maize Grain Yield. Agron J [Internet]. John Wiley & Sons, Ltd; 2006;98:1532–43. Available from: https://doi.org/10.2134/agronj2006.0038

19. Awal MA, Koshi H, Ikeda T. Radiation interception and use by maize/peanut intercrop canopy. Agric For Meteorol [Internet]. Elsevier; 2006 [cited 2019 Nov 21];139:74–83. Available from: https://www.sciencedirect.com/science/article/pii/S0168192306001523

20. Irmak S, Mutiibwa D. Dynamics of photosynthetic photon flux density and light extinction coefficient to assess radiant energy interactions for maize canopy. Trans ASABE. 2950 NILES RD, ST JOSEPH, MI 49085-9659 USA: AMER SOC AGRICULTURAL \& BIOLOGICAL ENGINEERS; 2008;51:1663–73.

21. Bergamaschi H, Dalmago GA, Bergonci JI, Krüger CAMB, Heckler BMM, Comiran F. Intercepted solar radiation by maize crops subjected to different tillage systems and water availability levels. Pesqui. Agropecuária Bras. scielo; 2010. p. 1331–41.

22. Vazin F, Hassanzadeh M, Madani A, Nassiri-Mahallati M, Nasri M. Modeling light interception and distribution in mixed canopy of common cocklebur (Xanthium stramarium) in competition with corn. Planta Daninha [Internet]. scielo; 2010;28:455–62. Available from: http://www.scielo.br/scielo.php?script=sci_arttext&pid=S0100-83582010000300001&nrm=iso

23. Gao Y, Duan A, Qiu X, Sun J, Zhang J, Liu H, et al. Distribution and Use Efficiency of Photosynthetically Active Radiation in Strip Intercropping of Maize and Soybean. Agron J [Internet]. John Wiley & Sons, Ltd; 2010;102:1149–57. Available from: https://doi.org/10.2134/agronj2009.0409

24. Ma DL, Xie RZ, Niu XK, Li SK, Long HL, Liu YE. Changes in the morphological traits of maize genotypes in China between the 1950s and 2000s. Eur J Agron. Elsevier; 2014;58:1–10.

25. Timlin DJ, Fleisher DH, Kemanian AR, Reddy VR. Plant Density and Leaf Area Index Effects on the Distribution of Light Transmittance to the Soil Surface in Maize. Agron J [Internet]. John Wiley & Sons, Ltd; 2014;106:1828–37. Available from: https://doi.org/10.2134/agronj14.0160

26. Wang Z, Zhao X, Wu P, He J, Chen X, Gao Y, et al. Radiation interception and utilization by wheat/maize strip intercropping systems. Agric For Meteorol [Internet]. 2015;204:58–66. Available from: https://www.sciencedirect.com/science/article/pii/S0168192315000325

27. Morales-Ruiz A, Loeza-Corte JM, Díaz-López E, Morales-Rosales EJ, Franco-Mora O, Mariezcurrena-Berasaín MD, et al. Efficiency on the Use of Radiation and Corn Yield under Three Densities of Sowing. Imhoff S, editor. Int J Agron [Internet]. Hindawi Publishing Corporation; 2016;2016:6959708. Available from: https://doi.org/10.1155/2016/6959708

28. Liu G, Hou P, Xie R, Ming B, Wang K, Xu W, et al. Canopy characteristics of high-yield maize with yield potential of 22.5Mgha−1. F Crop Res [Internet]. 2017;213:221–30. Available from: https://www.sciencedirect.com/science/article/pii/S0378429017307451

29. Liu X, Rahman T, Yang F, Song C, Yong T, Liu J, et al. PAR Interception and Utilization in Different Maize and Soybean Intercropping Patterns. PLoS One [Internet]. Public Library of Science; 2017;12:e0169218. Available from: https://doi.org/10.1371/journal.pone.0169218

30. Soleymani A. Light response of barley (Hordeum vulgare L.) and corn (Zea mays L.) as affected by drought stress, plant genotype and N fertilization. Biocatal Agric Biotechnol [Internet]. 2017;11:1–8. Available from: https://www.sciencedirect.com/science/article/pii/S1878818116304431

31. Dong W, Yu H, Zhang L, Wang R, Wang Q, Xue Q, et al. Asymmetric Ridge–Furrow and Film Cover Improves Plant Morphological Traits and Light Utilization in Rain-Fed Maize [Internet]. J. Meteorol. Res. 2018. p. 829–38. Available from: http://jmr.cmsjournal.net//article/id/c3c5ba66-bca7-4c87-86dc-394e49db7ee4

32. Li J, Xie RZ, Wang KR, Hou P, Ming B, Zhang GQ, et al. Response of canopy structure, light interception and grain yield to plant density in maize. J Agric Sci. 2018;156:785–94.

33. Perez RPA, Fournier C, Cabrera-Bosquet L, Artzet S, Pradal C, Brichet N, et al. Changes in the vertical distribution of leaf area enhanced light interception efficiency in maize over generations of selection. Plant Cell Environ. 2019;42:2105–19.

34. Kukal MS, Irmak S. Light interactions, use and efficiency in row crop canopies under optimal growth conditions. Agric For Meteorol [Internet]. 2020;284:107887. Available from: https://www.sciencedirect.com/science/article/pii/S0168192319305039
